# Operative management of fragility fractures of the pelvis – a systematic review

**DOI:** 10.1186/s12891-021-04579-w

**Published:** 2021-08-21

**Authors:** Daniel G. G. Wilson, Joshua Kelly, Mark Rickman

**Affiliations:** 1 Royal Adelaide Hospital, Port Road, Adelaide, SA 5000 Australia; 2 Present address: Brighton, UK; 3 Centre for Orthopaedic and Trauma Research, University of Adelaide, Adelaide, SA 5005 Australia

**Keywords:** Osteoporosis, Pelvic fracture, Pelvic ring, Fragility fracture of the pelvis, Percutaneous

## Abstract

**Background:**

The incidence of osteoporotic pelvic fractures in elderly patient is rising. This brings an increasing burden on health and social care systems as these injuries often lead to prolonged hospital admissions, loss of independence, morbidity and mortality. Some centres now advocate stabilisation of these injuries to reduce pain, facilitate early mobilisation, decrease hospital stay and restore independence. A systematic review of the literature was planned to establish the evidence for this intervention.

**Methods:**

A systematic review was performed according to PRISMA guidelines. A clinical librarian performed a search of the following databases: NHS Evidence, TRIP, the Cochrane Database of Systematic Reviews, MEDLINE and EMBASE. Seventeen eligible studies were identified with 766 patients.

**Results:**

The quality of evidence was poor with no good quality randomised trials. The majority of injuries were minimally displaced. Posterior ring injuries were most often stabilised with percutaneous screws which were sometimes augmented with void filler. A number of techniques were described for stabilisation of the anterior ring although fixation of the anterior ring was frequently not performed.

There was consistent evidence from the included studies that operative intervention significantly improved pain. Complications were minimal but there were increased failure rates when a single unaugmented sacroiliac joint screw was used. The limited availability of non-operative comparators made it difficult to draw firm conclusions about the efficacy of surgical over non-surgical management in these patients.

**Conclusions:**

Operative management of fragility fractures of the pelvis should be considered for patients failing a brief period of non-operative management, however prospective randomised trials need to be performed to provide improved evidence for this intervention. Surgeons should consider which fixation techniques for fragility fractures of the pelvis are robust enough to allow immediate weightbearing, whilst minimising operative morbidity and post-operative complications.

PROSPERO Systematic Review ID: CRD42020171237.

**Supplementary Information:**

The online version contains supplementary material available at 10.1186/s12891-021-04579-w.

## Background

The rising incidence of osteoporosis has brought significant challenges regarding the management of associated low energy injuries. In the past, high energy pelvic fractures associated with major trauma have predominated, however the incidence of low energy fragility fractures of the pelvis (FFP) is increasing and predicted to continue rising in the future [1–3].

High energy injuries often involve both the bony pelvis and associated ligaments and soft tissues. In contrast, low energy FFP are more commonly characterised by injuries affecting only the weaker osteoporotic bone. This observation is the basis for Rommen’s classification which divides FFP into four groups of increasing instability ranging from isolated pubic rami injuries to complex bilateral displaced sacral injuries [4].

Historically the management of FFP in the elderly has involved a brief period of bed rest and analgesia followed by return to mobilisation as pain allowed [5, 6]. Despite this, significant pain caused by the injury [7, 8] often leads to prolonged immobility [9]. The consequences of this in a vulnerable population are significant. The mortality rate is similar to hip fractures, there is significant associated morbidity, prolonged inpatient stay and patients often require temporary or permanent admission to nursing homes [3, 10–15]. As with other debilitating osteoporotic fractures, this injury carries with it a significant financial burden [16] and results in a significant reduction in patient reported quality of life [17].

Some centres now advocate surgical management of these injuries to improve outcomes [18, 19]. The aim of this systematic review is to evaluate evidence for surgical management of FFP. This includes fracture patterns encountered as well as fixation methods and timing of interventions. We also aimed to identify whether these interventions improve pain and other outcomes such as mobility, length of stay, quality of life and mortality compared to conservatively managed patients.

## Methods

This systematic review was registered with the International prospective register of systematic reviews (PROSPERO) Centre for Reviews and Dissemination, University of York and conducted in accordance with PRISMA guidelines [20]. A clinical librarian searched the followed databases; NHS Evidence, TRIP, the Cochrane Database of Systematic Reviews, MEDLINE and EMBASE. Bibliographic database searches were conducted using the NHS Evidence Healthcare Databases Advanced Search platform. The final search was conducted on 4th May 2020. Relevant natural language and controlled vocabulary terms were selected and combined, and final results reviewed. There were no language restrictions in the initial search, however studies not written in English were excluded.

All studies with fragility fractures of the pelvic ring including sacral insufficiency fractures were included in patients 60 years or older. Studies were excluded if they contained patients with high-energy injuries (defined as a fall greater than from standing height), or a pathological fracture from a cause other than osteoporosis. We also excluded patients managed with sacroplasty alone although included patients with augmentation of sacroiliac joint (SIJ) screws with bone cement or other void filler. Acetabular fractures and isolated iliac crest fractures were excluded as were studies with fewer than ten patients.

**Fig. 1:**
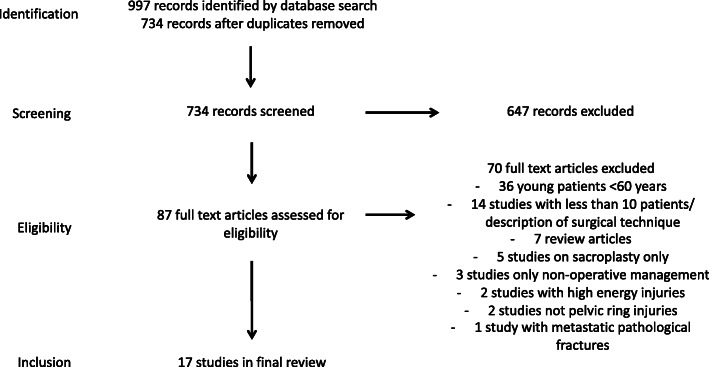
PRISMA flowchart of study selection

Abstracts were analysed by two authors (DW and JK). Relevant abstracts were selected for full text review and inclusion where relevant. Any disagreement on studies to include was decided by discussion and with the senior author (MR). A flowchart of the study selection can be seen in Fig. 1. Randomised and non-randomized studies were assessed for bias using The Cochrane Collaboration’s tool for assessing risk of bias [21, 22] and case series assessed by the Joanna Briggs Institute checklist for case series [23]. Studies were assessed by two authors (DW and JK) and any disagreements resolved by discussion with a third (MR). A narrative synthesis was then performed using these tools. Main outcomes were pain scores, quality of life, mobility, length of stay and mortality. Secondary outcomes were complications including re-operations, failure of fixation, neurological deficits, and infection. No meta-analysis was planned.

## Results

Seventeen studies were identified with 766 patients, of which 463 were managed operatively. One randomised study and three studies with non-operative comparison groups were identified. The remaining studies were case series. A summary of the results is presented in Table 1.

**Table 1:** Summary of results from included studies

Author, year and Study type	Patients	Classification and numbers	Indication for surgery	Fixation Method		Screw Augment	X-ray/CT	Post op weight bearing	Outcome measures	Results
				Posterior	Anterior					
Osterhoff et∼al. 2019 Retrospective case control [24]	230	“Low energy fractures of the pelvis”	Inability to mobilise 3–5 days post injury	2 SIJ screws - Unilateral = 33 bilateral = 24 Spinopelvic = 2 plate = 2.	Plate = 8 Ramus screw = 5 Infix = 4	–	X-ray	WBAT	1 and 2 year mortality. Majeed score	Overall 1 year mortality 21%. 23% early operative vs 17% non-operative *p* = 0.29. Majeed Score 66.1 operative vs 65.7 non-op (*p* = 0.91) @ 61 months average f/u. LoS 12 days operative vs 8 days non-op p = < 0.001
Balling et∼al. 2019 Randomised trial [25]	52	Rommens Type 2 = 52	Failed conservative management for 14 days	Minimum 2 transacral screws. 26 with sacroplasty, 26 without.	none	Additional sacroplasty in 26	CT	WBAT	VAS, ODI, Length of stay	Mean pre-op VAS SIJ 8.8 vs 9.0 SIJ + SP. Discharge VAS 3.5 and 3.6 respectively. P = < 0.05. No difference between groups. LoS 9.3 days SIJ vs 9.6 days SIJ + SP. Pre-op mean ODI SIJ 86.1 vs 86.2 SIJ + SP Decreased to 32.7 and 28.5 respectively at discharge.
Oikonomidis et∼al. 2019 Retrospective case series [26]	32	Rommens Type 1 = 1 Type 2 = 22 Type 3 = 9	Inability to mobilise after 1 week	Single SIJ screw = 31	Photodynamic bone stabilisation system = 32	–	X-ray	WBAT	VAS, length of stay, mortality	3% Mortality at 7.5 months. Mean discharge VAS 4.4, follow up VAS 3.0. Average LoS 16.5 days
Walker et∼al. 2018 Retrospective Cohort [27]	41	Young & Burgess LC1 = 26 “Sacral U” = 16	Inability to ambulate or severe pain on ambulation	Single transacral screw = 15, Double transacral screw = 1	none	–	X-ray	WBAT	VAS, LoS	VAS on admission operative group 7.4 improved to 3.5 on discharge, non-op 5.7 to 5.1. p = < 0.001 LoS 3.6 operative vs 4.2 non operative *P* = 0.51
Pulley et∼al. 2018 Retrospective case series [28]	16	“Sacral U” = 16	Failure of conservative management or inability to weight bear	2 transacral screws = 13, 2 SIJ screws bilaterally = 1, 1 transacral screw + 1 SIJ screw bilaterally = 1, 1 SIJ screw bilaterally = 1	none	–	X-ray	WBAT	VAS	Average improvement in pre to post op VAS 3.7 p = < 0.05
Hoch et∼al. 2017 (1) Retrospective Case series [6]	128	OTA B2.1 = 115 B3.3 = 13 Operative, non-operative and failed non-operative groups	Unable to mobilise after 3 days with adequate analgesia	Single SIJ screw = 28, 2 SIJ screws = 6, Bilateral SIJ screws = 14, Triangular fixation = 2.	Plate = 3	Additional sacroplasty in 13	CT in 7 cases	WBAT	VAS, SF12, LoS, Mortality, EQ. 5D	2 Year mortality 41% non-op vs 20% operative p = < 0.05. Mean LoS 18.1 operative vs 9.2 non-op P = < 0.001. Mean EQ. 5D at 2 years non-op 75.1, failed non-op 76.3, operative 74.6 - no significant difference. SF12 no difference between groups
Eckardt et∼al. 2017 Retrospective case series [29]	50	Rommens Type 2 = 15, Type 3 = 10, Type 4 = 25	persistent mobility limiting pain	Single screw = 37, 2 screws = 11, Plate = 2. transacral screws = 23, SIJ screws = 27	Plate = 14 (combined with single screw = 11, double screw = 1)	–	CT	WBAT	VAS, TUG, Mortality	1 year mortality 10%. TUG test at 2 years: 0–10s 5pts (16%), 10–20s 15pts (44%), 20–30s 7pts (22%), > 30s 6pts (19%). VAS at rest 0/10 20pts (61%), VAS 1–3 7pts (21%), VAS > 3 6pts (18%). Post TUG VAS 0/10 17pts (52%), VAS 1–3 6pts (18%) VAS > 3 10pts (30%)
Hoch et∼al. 2017 (2) Prospective case series [14]	34	Rommens Type 2 = 25 Type 4 = 8	Persistent immobilisation	Single SIJ = 25, Double SIJ = 1, Single SIJ bilaterally = 8	Plate = 16, Ramus screw = 16 Plate and screw = 1	All screws augmented with PMMA	X-ray	WBAT	VAS, SF12, LoS	VAS on admission 6.7 admission vs 2.7 day prior to discharge (p = < 0.001) SF12 at 1 year no difference to age matched controls. LoS 14 days
Sanders et∼al. 2016 Retrospective case series [30]	11	Bilateral sacral = 7 Jumpers variant = 1 unilateral sacral = 2, S1 stress = 1	Failure of non-operative measures or pain limiting mobilisation	1 transacral screw = 10, 2 transacral screws = 1	none	–	X-ray	WBAT	VAS, ODI, LoS, Mortality	1 year mortaility 10%. Mean VAS 9.1 pre-op vs 3.4 post-op and 2.4 final follow up P = < 0.01. Mean LoS 2.5 days, Mean ODI 71.6 pre-op to 17.6 post-op and 14.6 final follow up p = < 0.01
Collinge et∼al. 2016 Retrospective case series [8]	24	OTA B2.2 = 15, B3.3 = 8, C3.2 = 1	Acute fractures thought to be unstable or with marked pain limiting mobility	Single SIJ screw = 15, Single transacral screw = 9	none	All screws augmented with CaPO4	X-ray	WBAT	VAS	Mean VAS 7.9 pre-op vs 3.4 at discharge p = < 0.001. VAS 3.2 at 6 weeks, 2.0 at final follow up
Hopf et∼al. 2015 Retrospective case series [19]	30	Anterior and posterior = 18, Unilateral posterior = 1, Bilateral posterior = 11	Persistent pain or unacceptable mobility reduction after 6 days conservative management	Single SIJ = 6, Double SIJ = 12, Triple SIJ = 1, Bilateral SIJ = 2, Bilateral double SIJ = 6, Bilateral triple SIJ = 1, Bilateral SIJ - 2 screws one side, 1 screw other = 2,	none	–	X-ray	WBAT	VAS	Mean VAS 6.8 on admission, 6.0 after bed rest vs 2 days post op VAS 3.6 p = < 0.001, 1.8 on discharge p = < 0.001
Arduini et∼al. 2015 Retrospective case series [31]	14	Rommens Type 2 = 3, Type 3 = 9, Type 4 = 2	6 months failed conservative treatment	Single SIJ = 8, Plate and screw = 3, Spinopelvic fixation = 2	Screw = 2, plate = 3, ‘screw or plate in Rommens Type 3’ = 9	–	X-ray	Bed rest for 4–6 weeks	LoS	LoS 6 days post-op
Wahnert et∼al. 2013 Retrospective case series [32]	12	“insufficiency fractures” = 12	5–7 days conservative treatment without improvement	Single SIJ = 12	Infix = 3	All screws augmented with PMMA	CT	WBAT	VAS	Mean VAS 8.2 pre-op vs 2.6 post op (no statistical analysis performed)
Gansslen et∼al. 2013 Retrospective case series [7]	25	OTA B2.1 = 24, B3.3 = 1	not described	none	Supra-acetabular external fixator = 25	–	X-ray	Partial on side of injury	VAS, LoS	Mean VAS pre-op 7.7, Post-op VAS 2.3 (p = < 0.001), Frame removal (mean 4 weeks) VAS 0.6 p = < 0.0003. Average discharge 7 days post op
Mehling et∼al. 2012 Retrospective case series [18]	11	“insufficiency fractures”	fatigue fracture of sacrum or sacroiliac instability	Transacral bar = 11	ORIF = 3	–	X-ray	WBAT	German Multicentre Pelvis Study Group Score	German Multicentre Pelvis Study Group outcome score at 14 months - 7 point scale summarising radiological, clinical and social reintegration. 2 Excellent, 5 Good, 4 Fair
Lau et∼al. 2010 Retrospective case series [33]	37	Young & Burgess isolated pubic rami = 15, LC1 = 13, LC2 = 9	not described	Plate = 7	Screw = 1	–	X-ray	WBAT	Mortality	1 year mortality: 27% Rami fractures, 23% LC1, 13% LC2 (Operative intervention in 7/9 LC2 only)
Vanderschot et∼al. 2009 Retrospective case series [34]	19	Unilateral sacral = 1, Bilateral sacral = 18	not described	Transacral bar = 19	none	–	CT	WBAT	VAS	Mean VAS pre-op 6.8 to 2.3 at 9 months (p = < 0.001). LoS 3.6 op vs 4.2 non-op P = 0.51

*OTA* Orthopaedic Trauma Association, *LC1* Lateral Compression Type 1, *LC2* Lateral Compression Type 2, *SIJ* Sacroiliac Joint, *Infix* Internal fixator, *PMMA* Polymethylmethacrylate, *CT* Computed Tomography, *VAS* Visual anologue scale, *ODI* Oswestry Disability Index, *TUG* Timed up and go test, *LoS* Length of Stay, *WBAT* Weight bear as tolerated, *SP* Sacroplasty

### Quality assessment

Overall the quality of evidence for surgical management of FFP was low. The only randomised trial investigated the outcomes of FFP stabilised with transacral screw fixation with and without additional sacroplasty [25]. This non-blinded study also suffered from a high risk of selection bias, therefore drawing conclusions between the two groups was difficult. Despite this, robust follow-up and comprehensive reporting gave useful information regarding the pre and post-operative outcomes of surgically managed FFP.

The three comparative studies all suffered from high levels of bias. Groups were often poorly matched, had non-standardised interventions and suffered from incomplete follow up. All studies suffered selection bias as only patients in more significant pre-operative pain were selected for operative intervention. Whilst this may reflect a common scenario facing surgeons, drawing firm conclusions between the groups was difficult.

The quality of case series was variable. A common issue with the quality was a lack of pre-operative assessment of outcomes – particularly pain scores. Only six of the 13 case series contained pre-and post-operative/discharge pain scores for comparison. Most had some assessment of mobility or independence but these were often crude and unvalidated scoring systems and assessments that lacked a pre-operative comparison. Exclusion criteria, patient demographics and comorbidities were often poorly described or absent.

### Fracture patterns

A number of classification systems were used to classify injuries including the Orthopaedic Trauma Association (OTA) [35], Young and Burgess [36] and Rommen’s [4] classifications. Some studies did not utilise a recognised classification system and described fracture locations or grouped injuries as ‘sacral insufficiency’ fractures. This heterogeneity of fracture classifications made comparisons and analysis difficult between studies. Over 80% of the fractures studied consisted of minimally displaced unilateral or bilateral injuries suitable for percutaneous posterior fixation (Lateral compression type 1, Rommens Type II, OTA type B2). A minority of fractures were more displaced posterior unilateral fractures (5%), Displaced bilateral fractures (5%) and “Sacral U” type fractures (4%). This distribution is similar to those previously described [4, 37].

### Fixation methods

Numerous fixation methods were described for anterior and posterior ring injuries. Most patients underwent some form of posterior fixation. Only 26 (6%) patients had anterior fixation alone.

#### Posterior ring

**Fig. 2:**
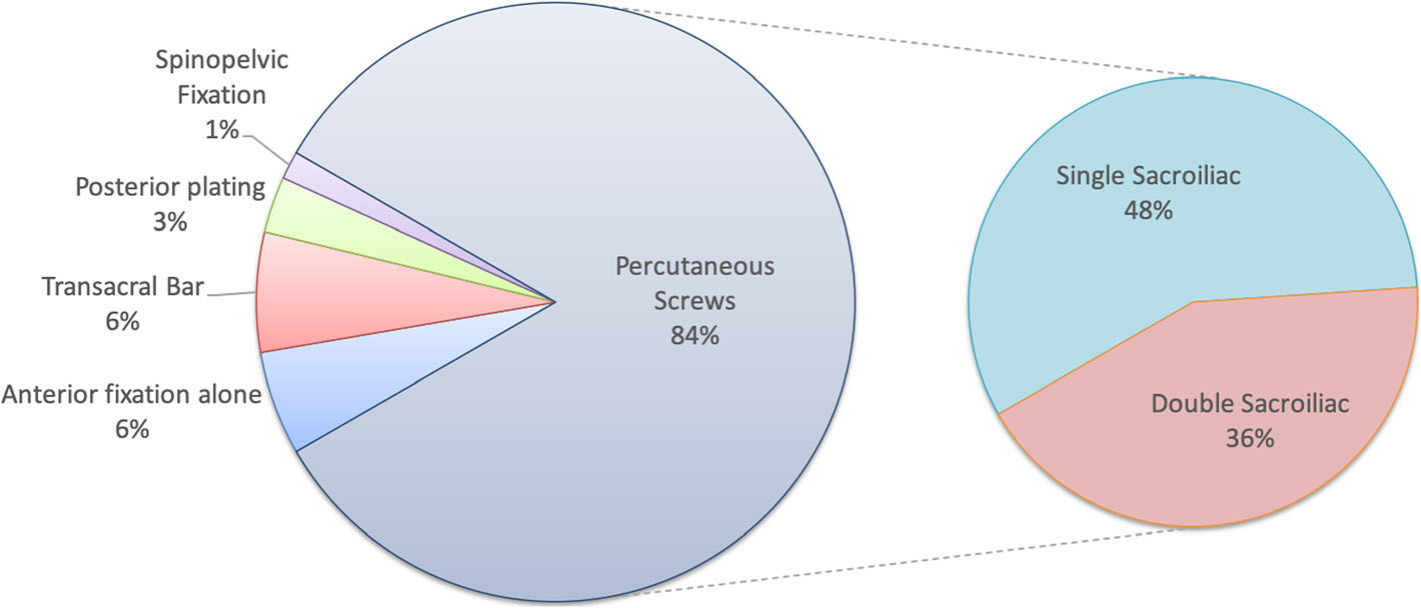
Breakdown of posterior fixation strategies

The majority of studies used percutaneous screws to fix posterior ring injuries. Figure 2 summarises the main methods of posterior fixation. 48% of patients had posterior stabilisation with a single screw whereas 36% had multiple screws. Most screws were SIJ screws crossing a single sacroiliac joint, however 23% of patients had longer transacral screws, passing from one side of the pelvis through both sacroiliac joints to the contralateral ilium [8, 25, 27–30]. 16% of patients received screw augmentation with polymethymethacralate (PMMA) or other void filler [8, 14, 32].

Around 25% of patients had a single, unaugmented SIJ screw. Eckardt examined reoperation rates for different screw configurations. They found a significantly increased reoperation risk when a single SIJ screw (not transacral or augmented) was used for posterior stabilisation regardless of whether the anterior ring was stabilised or not [29].

Two papers, with a total of 30 (6%) patients, described the use of a percutaneous transacral bar [18, 34] to treat bilateral sacral fractures.

There was consistent evidence that pain scores were reduced post-operatively with any percutaneous posterior ring fixation.

Seven patients in one study had plate fixation for their iliac fractures without SIJ screws [33]. Five patients in two studies had their percutaneous screw fixation augmented with a posterior plate [29, 31] and seven patients in three studies had spinopelvic fixation [14, 24, 31]. This method of fixation was reserved for the most complex and displaced fractures, which were not suitable for other less invasive methods of fixation.

#### Screw augmentation

Five studies used sacroplasty or screw augmentation. Balling et al. performed a prospective randomized study comparing a minimum of two transacral screws with and without PMMA sacroplasty. There was a statistically significant reduction in post-operative VAS scores and Oswestry Disability Index (ODI) scores in both groups but there was no difference between the groups.

Four other case series described screw augmentation. One utilised the technique in some patients but didn’t comment on indications or analyse these patients separately [14]. The others all showed statistically significant improvements in post-operative pain scores. No screw loosening or failure was described in any study [6, 8, 32].

#### Anterior fixation

Anterior fixation of the pelvic ring was variable. 322 (70%) operatively managed patients had no anterior fixation. Seven studies did not utilise any form of anterior fixation [8, 19, 25, 27, 28, 30, 34]. Some chose to stabilise all the anterior ring injuries with either percutaneous screws or an open reduction and plate fixation [31]. Other studies performed selective fixation for more displaced injuries [6, 14]. One case series used external fixation alone for 4 weeks with significant decrease in post-operative pain [7]. Two studies used an anterior internal fixator (Infix) on seven patients of [24, 32]. Another used a photodynamic bone stabilization system to stabilise rami fractures with concurrent posterior screw fixation [26]. Overall there was no consensus regarding method or indication for anterior fixation.

### Pain

Ten studies chose the Visual Analogue Scale (VAS) to quantify pain pre and post-operatively. Post-operative scores were either performed 24–48 h post-operatively or at discharge from hospital but in one case it was performed at an average of 9 months follow up [34]. All these studies in isolation showed a statistically significant improvement in average VAS scores. The average decrease in VAS amongst these studies was 4.5 which exceeds the minimally important clinical difference of 2.0 for patients with low back pain [38]. Only one study with a non-operative comparison group reported on pain pre-injury and on discharge. It showed a significant decrease in VAS from 7.4 pre-operatively to 3.5 in the operative group compared to the non-operative cohort who had an average VAS of 5.7 on admission and 5.1 on discharge. The operative patients had a significantly longer walking distance on discharge and 75% were discharged back home compared to 20% in the non-operative group [27].

Three papers reported VAS post operatively at various timepoints but with no pre-operative comparison. All of these showed low VAS scores, which were comparable to the scores of other studies [14, 26, 29].

### Disability/mobility outcomes

A broad selection of disability and quality of life scoring systems were used by authors. Two studies used the Oswestry Disability Index (ODI) questionnaire pre and post-operatively. The randomised study compared transacral screws with and without sacroplasty augmentation and showed a significant average improvement from 86.2 to 30.6 between pre and post-operative assessment but with no significant difference between the two cohorts [25]. One of the better quality case series also showed a significant average improvement from 71.6 to 17.6 after management with a single transacral screw [30]. SF-12 and EQ-5D scores were reported in two studies and found no difference between uninjured comparisons at 1 year [6] or operatively and non-operatively managed patients at 2 years [14].

One retrospective case control study performed Majeed scores at an average of 61 months post operatively and found no significant difference (66.1 operative, vs 65.7 non-op *p* = 0.91) [24].

Mehling et al. utilised a scoring system by the German Multicentre Pelvic Registry [39] which assesses radiographic, clinical and social reintegration domains. It reported 2 excellent, 5 good and 4 fair results at an average of 14 months follow up, but with no pre-operative comparison [18].

Most studies had an assessment of mobility or degree of post-operative independence. These are summarised in Table 2. Again there was significant heterogeneity in the method and timings of assessment, which made comparisons difficult. One of the cohort studies by Walker et al. showed significantly longer walking distances with operatively managed patients who were also more likely to be discharged home (75% vs 20%) compared to non-operatively managed patients [27].

**Table 2:** Mobility/Independence Outcomes

		Author/Year	Mobility/Independence
Osterhoff et al. 2019 [24]	36(24%) patients returned home in operative group compared to 19(23%) in non-operative group	Sanders et al. 2016 [30]	All patients returned to pre injury level of function at an average 625 days of follow up
Balling et al. 2019 [25]	All patients discharged when able to mobilise upstairs	Collinge et al. 2016 [8]	Not described
Oikonomidis et al. 2019 [26]	Mobility at discharge: 10(31%) crutches, 21(66%) walker/rollator, 1 (3%) unable to mobilise. Follow up at 7 months: 11(34%) independent, 7(22%) crutches, 6(19%) walker, 1(3%) immobile	Hopf et al. 2015 [19]	Complete mobility regained in 73% at discharge
Walker et al. 2018 [27]	75% patients discharged home in operative group compared to 20% in non-operative group (p = < 0.001). Significantly longer walking distance in operative vs non-operative at discharge (95.4 vs 35.2 ft p = < 0.01)	Arduini et al. 2015 [31]	Bed rest 4–6 weeks post op. At 6 months 11 patients (79%) had normal mobility, 1(7%) single crutch, 1 (7%) two crutches
Pulley et al. 2018 [28]	Average day 1 mobilisation 102 ft	Wahnert et al. 2013 [32]	All patients could be mobilised to their pre-operative levels
Hoch et al. 2017 (1) [6]	Not described	Gansslen et al. 2013 [7]	24 patients full mobility pre injury and 1 patient mobile with walker. At discharge 14 (58%) regained full mobility. 7 (28%) partial weight bearing. 21 (88%) patients regained baseline mobility at frame removal (average 4 weeks)
Eckardt et al. 2017 [29]	73% independent at home, 13% lost independence. 60% Performed TUG test <30s	Mehling et al. 2012 [18]	Not described
Hoch et al. 2017 (2) [14]	At 1 year 9(26%) patients mobile without aids, 6(18%) required 1 or 2 crutches, 12 (35%) with walker	Lau et al. 2010 [33]	At 3 months 53% baseline mobility isolated rami fractures, 62% LC1 and 56% LC2 fractures
		Vanderschot et al. 209 [34]	5 point ADL score used. Average 3.26 pre op improved to 1.68 at follow up (P = < 0.0001) at an average of 9 months

*LC1* Lateral Compression Type 1, *LC2* Lateral compression type 2, *ADL* Activities of Daily Living

### Length of stay

Walker et al. noted no significant difference in the length of stay between operative and non-operative groups (3.6 vs 4.2 days respectively) [27]. Hoch noted a significantly greater length of stay in operatively treated patients (18 days vs 9 days), although this did include an average of 6 days for an attempt at conservative management pre operatively [14]. Length of stay varied from an average of 2.5 to 16 days in the remaining studies reporting this outcome.

### Mortality

Hoch et al. studied mortality rates at 2 years in three cohorts. They noted that 2-year mortality was significantly greater (41%) in the non-operative group compared to the operative and failed non-operative groups (18 and 21% respectively) although the operative group was noted to be younger on average [14]. Another study comparing operative and non-operative groups showed no significant difference in mortality at 1 year (23% vs 17% *p* = 0.29). It did however, show a survival benefit in the operative group develop after 2 years [24]. Reported 1 year mortality rates ranged from 10 to 27% in other studies.

### Complications

Table 3 summarises the complications reported in the included studies. Operative complications were relatively uncommon. The main reasons for reoperation were for symptomatic screw loosening, incorrect screw placement with neurological symptoms and evacuation of post-operative haematoma. One study noted a significant rate of symptomatic screw loosening of 18% where a single percutaneous sacroiliac joint screw was used compared to two screws [29]. Two studies utilizing screw augmentation described a PMMA leak. In one paper there were 4 cases of PMMA extrusion, 2 into the spinal canal but with no symptoms [25]. Another noted asymptomatic PMMA leakage in 3(8%) of cases [6].

**Table 3:** Complications and reoperations

	Reoperations (%)	Indications	Other
Osterhoff et∼al. 2019 [24]	No reoperations reported	–	In hospital complications more common in operative group (35%) vs non-operative group (14%) p = < 0.05
Balling et∼al. 2019 [25]	1 (2%)	evacuation post op haematoma	1 guidewire perforation into spinal canal, 4 cases cement extrusion - 2 into canal, 2 into soft tissue. All asymptomatic
Oikonomidis et∼al. 2019 [26]	1 (3%)	Implant failure requiring removal	2 pneumonia, 4 UTI
Walker et∼al. 2018 [27]	No reoperations reported	–	1 GI bleed in operative group. 1 GI bleed and 2 COPD exacerbations in non-operative group.
Pulley et∼al. 2018 [28]	No reoperations reported	–	no complications
Hoch et∼al. 2017 (1) [6]	4 (13%)	3 screw malposition, 1 revision for infection	2 patients (4%) required transfusion post op. Medical complications 8% non-operative group, 18% operative group
Eckardt et∼al. 2017 [29]	13 (26%)	1 screw malposition, 1 infection, 9 for symptomatic implant loosening, 2 patients revised for implant loosening required further revision for non-union	–
Hoch et∼al. 2017 (2) [14]	2 (6%)	1 screw malposition, 1 evacuation haematoma	3 asymptomatic PMMA leaks, 1 Pulmonary embolism
Sanders et∼al. 2016 [30]	No reoperations reported	–	No complications
Collinge et∼al. 2016 [8]	No reoperations reported	–	1 extravasation of calcium phosphate into sacroiliac joint - asymptomatic
Hopf et∼al. 2015 [19]	3 (10%)	1 screw malposition, 2 evacuation haematoma	1 intraoperative bleed requiring 3 units blood. 2 cases HAP and 2 Cases UTI in 3 patients
Arduini et∼al. 2015 [31]	No reoperations reported	–	1 intrapelvic iliac screw from spinopelvic fixation - asymptomatic and left in situ
Wahnert et∼al. 2013 [32]	No reoperations reported	–	No complications
Gansslen et∼al. 2013 [7]	No unplanned reoperations reported	All patients required planned removal of external fixator in outpatient setting	2 pin site infections managed with antibiotics. 1 pin loosening.
Mehling et∼al. 2012 [18]	No reoperations reported	–	1 temporary L5 nerve palsy
Lau et∼al. 2010 [33]	No reoperations reported	–	1 fibrous non-union. 1 permanent L5 nerve palsy
Vanderschot et∼al. 2009 [34]	2 (11%)	2 evacuation post op haematoma	–

*UTI* Urinary Tract Infection, *GI* Gastrointestinal, *COPD* Chronic Obstructive Pulmonary Disease, *PMMA* Polymethylmethacrylate, *HAP* Hospital Acquired Pneumonia

## Discussion

Traditionally FFP have been managed non-operatively, often with a period of bed rest with analgesia and then mobilisation [5, 40]. Knowledge regarding the detrimental effect of bed rest and immobility in the elderly population has been present for decades [41]. Even short periods of immobilisation or decreased mobility lead to prolonged loss of muscle strength [42]. As a consequence there is reduced capacity to perform activities of daily living leading to loss of independence [43, 44]. Mortality from non-operatively managed pelvic fractures is also similar to matched patients treated for hip fracture [45].

Elderly patients are unable to comply with restricted weightbearing regimes [46] – any management strategy should facilitate immediate full weight bearing with sufficient control of pain. This review highlights that whilst the quality of evidence for surgical fixation of FFP is poor, there is consistent data to support the idea that the majority of fracture patterns can be stabilised through minimally invasive methods, with a consequent reduction in pain. Whether this assertion is correct or if it translates to outcomes with regards mobility, length of stay, quality of life and mortality needs further investigation.

A heterogenous selection of fracture classifications were used by the studies, which made comparisons difficult. FFP often do not fit well into the OTA, Tile or Young and Burgess classifications as elderly patients tend to have bony injuries rather than ligamentous involvement [4, 8]. Recognising this, the Rommen’s classification was developed specifically for these injuries and may be more useful in classifying these injuries in the future [47].

The decision to pursue operative intervention in most of the studies was made after failure of conservative measures rather than based on fracture morphology. Whilst some unstable fractures may mandate surgical intervention, the commonest minimally displaced posterior injuries and even some more extensive and displaced injuries may be managed successfully with conservative measures. Conservative management was normally instituted for a period of 3–7 days although it did range up to 6 months. Given that relatively short periods of immobility can lead to significant morbidity we would suggest considering intervention after 48 h of failed conservative management and ideally within 1 week to avoid complications associated with prolonged immobility.

With regard to fixation techniques, when stabilising the posterior ring, the majority of authors utilised percutaneous posterior fixation where possible. Biomechanical studies have shown that two SIJ screws, a single augmented SIJ screw and a single transacral screw offer similar levels of stability and are all superior to single SIJ screw fixation in osteoporotic models [48–52]. There is some clinical evidence identified in this review that highlights a significantly increased rate of screw loosening when one unaugmented SIJ screw was used in isolation [29]. This finding was independent of whether or not the anterior ring was stabilised. We would suggest that a single un-augmented SIJ screw may not provide sufficient stability in the osteoporotic patient to facilitate early full weightbearing and adequate pain relief. Either multiple screws, longer transacral screws, or screw augmentation should be considered. Surgeons should be mindful of the potential additional risks and benefits of each option.

Supplementary anterior fixation was sporadic if present and there was no correlation between lack of anterior fixation and overall fixation failure. Tile noted that the posterior ring contributes around 60% to pelvic stability [53] and Matta confirmed that even in unstable pelvic injuries rami fractures did not require stabilization by internal or external fixation when the posterior ring was stabilised [54]. The data presented here would support the assertion that posterior fixation in these injuries is more critical than anterior fixation. Stabilising the anterior ring contributes to overall pelvic stability and this may understandably be desirable to surgeons managing osteoporotic FFP. Percutaneous screw fixation of the ramus is an attractive option in minimally displaced fractures, however there is some biomechanical evidence in osteoporotic bone that plate fixation is superior to percutaneous retrograde screw fixation, the trade-off being that this requires an open approach [55]. External fixation is a quick and relatively easy technique but its use in the osteoporotic patient raises concerns with regard to pin-site infection, loosening and patient acceptance [4]. The Infix is a newer development and current trials are ongoing to identify whether it is a suitable method for stabilising osteoporotic type 1 lateral compression fractures [56], however its use is also not without complications [57]. A large study of the German pelvic database comparing Tile B and C type pelvic fractures with the anterior component involving the obturator foramen showed a higher rate of complications from more extensive anterior and posterior surgery compared to posterior stabilization alone with no difference in fixation failure or mortality [58]. When electing to stabilise FFP, surgeons should be mindful that supplementary anterior fixation may not be necessary with a robust posterior fixation. If electing to stabilise the anterior ring, surgeons should be mindful of potential complications associated with the chosen method. Anterior fixation alone may be appropriate depending on the strength of fixation and degree of posterior instability [7].

Observed complications from surgical interventions were low. Most studies used either computed tomography (CT) guidance or fluoroscopy to insert screws. Incorrect screw placement was noted with both techniques but was infrequent and any iatrogenic neurological deficits resolved after removal and repositioning [6, 14, 19, 29].

We noted a previous systematic review of four studies on the effectiveness of surgical fixation of osteoporotic LC1 fractures. This found insufficient evidence to support guidance on the most effective treatment for patients sustaining this injury however where reported, mobility and function did improve after surgery [59]. This systematic review is a more comprehensive overview of 17 studies encompassing all types of FFP.

This systematic review does have some weaknesses. Firstly there was a lack of good quality randomised studies on which to base conclusions and most studies suffered from significant risk of bias. There was also significant heterogeneity in fracture classification and outcome measures. This made appropriate data pooling and analysis between studies difficult. We also excluded trials with less than 10 patients and excluded papers where an English translation could not be obtained. This only resulted in the loss of a small number of cases and therefore was unlikely to change the overall impression of the review.

## Conclusion

This systematic review set out to identify the evidence for surgical management of FFP. Overall the quality of evidence was low. Of the 17 studies identified only one was randomised but with high risk of bias. Three studies were identified with a non-operative comparator group but all suffered from significant bias. Despite this, consistent improvement in pain and mobility was noted with stabilisation of the pelvis, which was most often performed percutaneously to the posterior ring. Anterior fixation of the pelvic ring was often absent and variable techniques were used when present. More than one SIJ screw posteriorly, longer transacral screws or screw augmentation offer more robust fixation than single SIJ screws for stabilising the pelvis, especially in the context of osteoporosis. Surgeons should consider operative stabilisation of FFP after a brief period of conservative management to avoid morbidity associated with immobility.

## Supplementary Information

**Additional file 1.** Example of EMBASE search strategy.

## Data Availability

The datasets analysed and/or created where not presented are available from the corresponding author on reasonable request.

## Abbreviations

FFP: Fragility fracture of the pelvis
PROSPERO: International prospective register of systematic reviews
PRISMA: Preferred reporting instructions for systematic review and meta-analysis
NHS: National Health Service
SIJ: Sacroiliac Joint
OTA: Orthopaedic Trauma Association
PMMA: Polymethylmethacrylate
VAS: Visual analogue scale
ODI: Oswestry Disability Index
CT: Computed tomography
