# Supplementary material for: Operative management of fragility fractures of the pelvis – a systematic review
Source: BMC Musculoskelet Disord. 2021 Aug 21;22:717. doi: 10.1186/s12891-021-04579-w (PMC8380328; doi:10.1186/s12891-021-04579-w)
Supplement: Supplementary file 1 — Additional file 1. Example of EMBASE search strategy. [file 12891_2021_4579_MOESM1_ESM.docx]

EMBASE search strategy

1     *pelvis fracture/ (3223)
2     ((pelvis or pelvic) adj2 (fractur* or injur*)).ti,ab. (6763)
3     ((sacrum or sacral) adj2 (fractur* or injur*)).ti,ab. (1270)
4     (("pubic ramus" or "pubic rami") adj2 (fractur* or injur*)).ti,ab. (193)
5     ((fragility or geriatric or insufficiency) adj2 fractur* adj2 pelvi*).ti,ab. (152)
6     (osteopor* adj2 fractur* adj2 pelvi*).ti,ab. (27)
7     *fragility fracture/ (6400)
8     1 and 7 (52)
9     1 or 2 or 3 or 4 or 5 or 6 or 8 (8538)
10     exp *aged/ (48896)
11     (geriatric* or elder* or ageing or aging).ti,ab. (603746)
12     10 or 11 (616335)
13     9 and 12 (443)
